# Pericapsular Nerve Group Block and Iliopsoas Plane Block: A Scoping Review of Quadriceps Weakness after Two Proclaimed Motor-Sparing Hip Blocks

**DOI:** 10.3390/healthcare10081565

**Published:** 2022-08-18

**Authors:** Shang-Ru Yeoh, Yen Chou, Shun-Ming Chan, Jin-De Hou, Jui-An Lin

**Affiliations:** 1 Department of Anesthesiology, Wan Fang Hospital, Taipei Medical University, Taipei 116, Taiwan; 2 Center for Regional Anesthesia and Pain Medicine, Wan Fang Hospital, Taipei Medical University, Taipei 116, Taiwan; 3 Department of Medical Imaging, Far Eastern Memorial Hospital, New Taipei City 220, Taiwan; 4 Department of Anesthesiology, Tri-Service General Hospital and National Defense Medical Center, Taipei 11490, Taiwan; 5 Department of Anesthesiology, School of Medicine, National Defense Medical Center, Taipei 11490, Taiwan; 6 Division of Anesthesiology, Hualien Armed Forces General Hospital, Hualien 97144, Taiwan; 7 Center for Regional Anesthesia and Pain Medicine, Chung Shan Medical University Hospital, Taichung 40201, Taiwan; 8 Department of Anesthesiology, School of Medicine, Chung Shan Medical University, Taichung 40201, Taiwan; 9 Department of Anesthesiology, Chung Shan Medical University Hospital, Taichung 40201, Taiwan; 10 Department of Anesthesiology, School of Medicine, College of Medicine, Taipei Medical University, Taipei 110, Taiwan; 11 Pain Research Center, Wan Fang Hospital, Taipei Medical University, Taipei 116, Taiwan

**Keywords:** anterior hip capsule, iliopsoas plane, interfascial plane block, motor-sparing, pericapsular nerve group, PENG block

## Abstract

Iliopsoas plane (IP) is a fascial plane deep to the iliopsoas complex that can serve as a potential space for the injection of local anesthetics to selectively block the articular branches of femoral nerve and accessory obturator nerve to the anterior hip capsule. Two highly similar ultrasound-guided interfascial plane blocks that target the IP, pericapsular nerve group (PENG) block and iliopsoas plane block (IPB), were both designed to achieve motor-sparing sensory block to the anterior hip capsule. However, the most recent evidence shows that PENG block can cause 25% or more of quadriceps weakness, while IPB remains the hip block that can preserve quadriceps strength. In this scoping review of quadriceps weakness after PENG block and IPB, we first performed a focused review on the complicated anatomy surrounding the anterior hip capsule. Then, we systematically searched for all currently available cadaveric and clinical studies utilizing PENG block and IPB, with a focus on quadriceps weakness and its potential mechanism from the perspectives of fascial plane spread along and outside of the IP. We conclude that quadriceps weakness after PENG block, which places its needle tip directly deep to iliopsoas tendon (IT), may be the result of iliopectineal bursal injection. The incidental bursal injection, which can be observed on ultrasound as a medial fascial plane spread, can cause bursal rupture/puncture and an anteromedial extra-IP spread to involve the femoral nerve proper within fascia iliaca compartment (FIC). In comparison, IPB places its needle tip lateral to IT and injects just one-fourth of the volume of PENG block. The current evidence, albeit still limited, supports IPB as the true motor-sparing hip block. To avoid quadriceps weakness after PENG block, a more laterally placed needle tip, away from the undersurface of IT, and a reduction in injection volume should be considered. Future studies should focus on comparing the analgesic effects and quadriceps function impairment between PENG block and IPB.

## 1. Introduction

Pericapsular nerve group (PENG) block is a novel interfascial plane block targeting the articular branches of femoral, obturator, and accessory obturator nerve to the anterior hip capsule [1]. Since its publication in 2018, it has become a highly popular ultrasound-guided regional technique as a proclaimed motor-sparing hip block among the anesthesiologists and emergency physicians. However, quadriceps weakness after PENG block was soon reported [2,3], and some recent randomized controlled trials have revealed an alarmingly high frequency of post-operative quadriceps weakness [4,5]. The exact mechanism of femoral nerve proper involvement after PENG block is still speculative and was thought to result from local anesthetics spreading superficially either via a plane between pectineus and psoas major or intramuscularly [6]. A highly similar technique called the iliopsoas plane block (IPB) [7,8], despite still being largely underutilized clinically, may provide essential information regarding the spread of injectate to undesired neural targets after PENG block. To approximate the true motor-sparing hip block, IPB should therefore be brought to receive more clinical attention. 

As Marhofer et al. [9] succinctly put it, regional anesthesia is applied anatomy. The key to understand how an interfascial plane block works (and fails) will always be the detailed neuromusculofascial anatomy relevant to that specific block. Since the anterior hip is an anatomically complicated area, it is not surprising that most clinical practitioners using PENG block are still in general unfamiliar with it. Therefore, in view of PENG block’s burgeoning popularity in clinical practice and the recent upsurge of interests in IPB, a focused review of the musculofascial anatomy surrounding the anterior hip capsule, followed by a synthesis with the currently available evidence and its clinical implications to the motor-sparing property of PENG block and IPB, is warranted. 

In this scoping review on the quadriceps function after PENG block and IPB, we aim to bridge the anatomical knowledge gap of hip for clinical practitioners, clarify the similarities and differences between PENG block and IPB, and to scope out future research di-rection on this topic. We will first provide an anatomical framework of the important structures surrounding the anterior hip capsule to lay out a foundation for discussion. Then with a focus on the final needle tip position and the pattern of injectate spread, we investigated systematically on the cadaveric and clinical studies utilizing PENG block and IPB with an attempt to provide technical hints for a true motor-sparing block to the anterior hip capsule.

## 2. Applied Anatomy

### 2.1. Iliopsoas Complex

**Figure 1:**
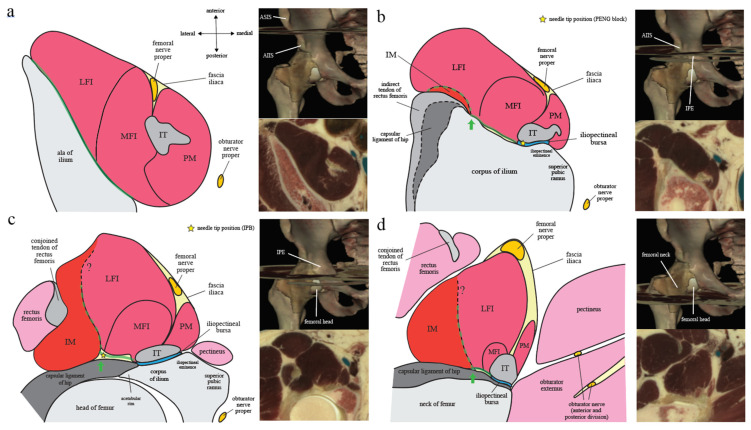
The iliopsoas complex and its surrounding structures of a human right hip were illustrated in transverse sections according to the real cadaveric images (right lower panels) at four successive levels (right upper panels) in craniocaudal order: (**a**) At the level between anterior superior iliac spine (ASIS) and anterior inferior iliac spine (AIIS): The posterolateral wall of iliopsoas plane (IP) is composed entirely of an osseous surface, which is the iliac ala (green solid stroke); (**b**) At the level of iliopectineal eminence, i.e., the pericapsular nerve group (PENG) block level: Note that as IM emerges, IP starts to divide into a muscular part (green dashed stroke), which is the raphe between IM and lateral fibers of iliacus (LFI), and an osseous part (green solid stroke), which is the iliac corpus; (**c**) At the level where femoral head dives deep into the acetabular rim, i.e., the iliopsoas plane block (IPB) level: As the capsular ligaments of hip extend inferolaterally from the acetabular rim, the ligamentous IP, which is a potential space between capsular ligament of hip and the iliopsoas complex, gradually replaces the osseous IP as its posterior wall (green solid stroke). And as the IM muscle substance becomes bigger at this level, rendering the muscular IP (green dashed stroke) more vertical, IP becomes L-shaped; (**d**) At the level where femoral head transits into femoral neck: IP remains L-shaped but the ligamentous IP becomes smaller in area as IM stretches inferomedially, closing up the gap between itself and IT, and inserts into femur just distal to lesser trochanter. At this level, the two divisions of obturator nerve have just left the obturator canal, with the anterior branch traveling inside subpectineal plane (SP) and the posterior branch passing through the obturator externus muscle, on its way to the SP. The most superficial part of the raphe between iliopsoas complex and IM could not be readily distinguished on the cadaveric images and are therefore drawn as a dashed line indicated with question mark. The green arrows indicate the junction between the osseous or ligamentous IP and the muscular IP, and the yellow stars mark the respective final needle tip position of PENG block (**b**) and IPB (**c**). Cadaveric images were reconstructed from real cadavers by the Visible Human Project® of National Library of Medicine and excerpted from the VH Dissector with permission from Touch of Life Technologies Inc (www.toltech.net). IT: iliopsoas tendon, MFI: medial fibers of iliacus, PM: psoas major muscle fibers.

Iliopsoas muscle is an elongated and complex musculotendinous unit that originates superiorly from the lumbar vertebrae and iliac crest and inserts inferiorly to the lesser trochanter and the infratrochanteric ridge [10]. It is composed of several musculotendinous components of different arrangement dependent on anatomical levels [10]. Between the lower portion of iliac ala and femoral neck, four major components can be observed in transverse sections (from lateral to medial): lateral muscle fibers of iliacus (LFI), medial muscle fibers of iliacus (MFI), iliopsoas tendon (IT), and psoas major muscle fibers (PM) [11] (Figure 1). IT is a conjoined tendon fused by the lateral psoas major tendon and the medial iliacus tendon [12]. 

From the level of anterior inferior iliac spine (AIIS) going caudally, a muscle closely associated with the iliopsoas complex named iliacus minor (IM, or called ilioinfratrochanteric or iliocapsular muscle) appears deep and then lateral to the iliopsoas complex and is consistently demarcated by a connective tissue raphe from the LFI (Figure 1b–d) [10,11,13]. The IM muscle has its own origin and insertion that are independent from its closely associated iliopsoas complex [13]. Its main origin is an elongated attachment that is strongly attached to the anteromedial hip capsule overlying the anterior surface of fem-oral head, while its secondary origin is the inferior border of AIIS [13]. IM muscle can be-come hypertrophied and more prominent in a dysplastic hip [14]. 

The intrapelvic part of iliopsoas complex makes a 40–45 degree angle over the ventral edge of the iliac bony concave situated between AIIS and iliopectineal eminence before its extrapelvic (femoral) part inserts deep into the lesser trochanter of femur [12]. At this level, the iliopsoas complex becomes bordered medially by the pectineus muscle and laterally by IM (Figure 1c,d).

### 2.2. Iliopectineal Bursa

Iliopectineal bursa is the largest bursa in human body that is situated deep to the musculotendinous portion of the iliopsoas complex [15]. Due to its close relationship with the tendinous part of iliopsoas complex, it is also called the subtendinous iliac (psoas) bursa [16]. The size of bursa was found to vary widely, with the majority ranged between 2–4 cm in width and 5–7 cm in length [17]. Proximally, the synovial bursa lies on the iliopectineal eminence [18] (Figure 1b) and in some cases can extend intrapelvically (cranially) over the brim of pelvis [17]. Distally, the bursa passes across the front of hip capsule (Figure 1d) and extends almost as far as to the lesser trochanter [18]. Normally, iliopectineal bursa is in an immediate relationship to IT [18]. Besides, direct insertions between MFI and iliopectineal bursa also exist, and identification of the bursa can be used to locate iliopectineal eminence [13]. In healthy individuals, the iliopectineal bursa contains a small amount of synovial fluid that is not easily visualized on ultrasound [19]. However, when disturbed by pathological processes, it distends anteriorly and can often be seen as an anechoic or hypoechoic sac medial to the iliopsoas complex [20,21]. Around 15% of the iliopectineal bursa were found to be communicated with the synovial sac of the hip joint as a product of attrition [17], via a circular aperture between the pubofemoral ligament and the descending (medial) part of iliofemoral ligament [16]. 

According to these anatomical features of iliopectineal bursa, the final needle tip position of PENG block, as originally described by Giron-Arango et al. [1], would frequently end up inside the iliopectineal bursa (Figure 1b). In fact, although it was never explicitly explained by the group, PENG block might be deliberately designed to target the bursa for pericapsular coverage [22,23].

### 2.3. Iliopsoas Plane

**Figure 2:**
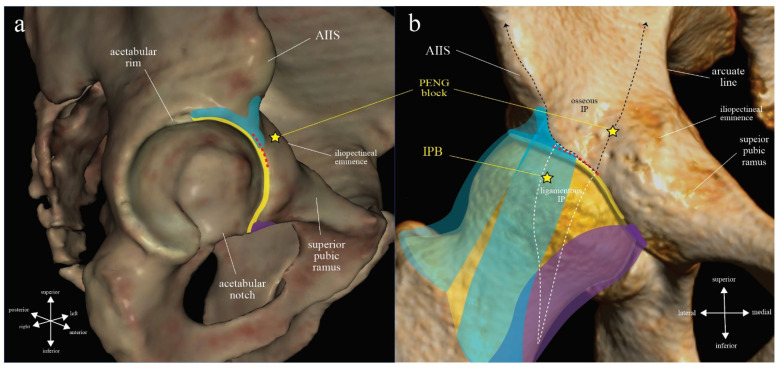
Capsular ligaments of hip of the anterior hip capsule and distinction of the osseous and ligamentous iliopsoas plane (IP): (**a**) Lateral view of the right-sided acetabulum, with femur re-moved, is shown here to demonstrate the proximal attachments of the capsular ligaments of hip of anterior hip capsule and their spatial relationship to the acetabulum and its surrounding iliopubic structures. The attachments of iliofemoral ligament, the capsular fibers, and pubofemoral ligament to the acetabular rim are colored in blue, yellow, and purple, respectively. Both iliofemoral ligament and pubofemoral ligament are distinct thickening of the capsular fibers. The figure was modified from a reconstructed cadaveric image by the Visible Human Project® of National Library of Medicine acquired from the VH Dissector with permission from Touch of Life Technologies Inc (www.toltech.net); (**b**) In this three-dimensional computed tomography image of the right-sided hip joint reconstructed from a real patient with volume-rendering technique, the osseous IP refers to the wide shallow groove between anterior inferior iliac spine (AIIS) and iliopectineal eminence and its cranial extension on ilium, as demarcated by the arrowed dashed black line. Iliopsoas valley, marked as the dashed red line (also in Figure 2a), is a depression of the anterior acetabular rim that is continuous with the AIIS-iliopectineal eminence concave to allow passage of the iliopsoas complex inferiorly over the femoral head. Capsular ligaments of hip are illustrated as colored bands connecting the acetabular rim and the intertrochanteric line of femur, with the same colors coded as in Figure 2a. The blue-colored iliofemoral ligament has a transverse (lateral) part and a descending (medial) part. The ligamentous IP is demarcated by the dashed white line and refers to the fascial plane between iliopsoas complex and fibers of the capsular ligaments of hip, with iliopsoas tendon (IT, not shown) as its medial border and iliacus minor muscle (IM, not shown) as its lateral border. The yellow stars mark the final needle tip positions of pericapsular nerve group (PENG) block and iliopsoas plane block (IPB).

Iliopsoas plane (IP) is a fascial plane originally coined by Nielsen et al. to describe the pattern of injectate spread after IPB [7,8]. Its anteromedial wall was defined as the extrapelvic part of iliopsoas muscle with intrapelvic origin, while its posterolateral wall was divided into a cranial part and a caudal part by the AIIS. Cranial to AIIS, the posterolateral wall of IP was defined as the iliac corpus and ala (Figure 1a,b). While caudal to AIIS, its posterolateral wall becomes the IM muscle and its associated descending (medial) part of iliofemoral ligament, which is a component of the capsular ligaments of hip (Figure 1c,d). However, the final needle tip position of PENG block is caudal to AIIS and on the iliac corpus at the same time (Figure 2). In order to put PENG block into IP’s context, we redefined the anatomical landmark used for the craniocaudal division of IP’s posterolateral wall from AIIS alone to the anterior acetabular ridge between AIIS and iliopectineal eminence (psoas valley) (Figure 2).

#### 2.3.1. Iliopectineal Eminence

Iliopectineal eminence is a bony protuberance connecting iliac arcuate line and pubic crest that marks the union of ilium and pubis [16]. On the iliac corpus proximal to the anterior acetabular rim, a wide shallow groove is bounded laterally by AIIS and medially by iliopectineal eminence [16] (Figure 2). The groove is occupied by the converging fibers of iliacus muscle laterally and IT medially, with iliopectineal bursa lying underneath the IT [16]. The groove connects inferiorly with a depression on the anterior acetabular rim called psoas valley, which provides passage for IT and changes its direction just as a pulley changes the course of a cord [24] (Figure 2).

#### 2.3.2. Capsular Ligaments of Hip: Iliofemoral Ligament

Capsular ligaments of hip are distinct thickening of the capsular fibers to reinforce the hip capsule and are comprised by several ligamentous complex (Figure 2) [25]. Among them, only the descending (medial) part of iliofemoral ligament is of relevance to IPB. 

The articular capsule of hip consists of strong and dense fibers, with various thick-ness according to the location, that connect the margins of the acetabulum to proximal femur [25]. Proximally, it is widely attached to the osseous margin of acetabulum just be-yond the labrum and is continuous with the periosteum of acetabulum [25] (Figure 2). Anteriorly, the capsule is thick and has predominately longitudinally oriented fibers related to the iliofemoral ligament [25]. 

Iliofemoral ligament originates proximally from the base of AIIS and the iliac portion of the acetabular margin [25] and is highly variable in the location of its acetabular origin [26]. It spreads distally on the anterosuperior region of hip joint, in an inverted Y, and is composed of a transverse (lateral) and a descending (medial) part [16]. Iliofemoral ligament can become thickened and distorted in hips with pathology [27].

#### 2.3.3. Iliopsoas Plane (IP): *Osseous*, *Ligamentous*, and *Muscular*

Based on the musculoskeletal anatomy as described above, we divide Nielsen et al.’s IP into three parts. The osseous and ligamentous part are named after their respective an-atomical floor on which iliopsoas complex lies (Figure 2), while the muscular part refers to the raphe between the iliopsoas complex and its laterally associated IM muscle (Figure 1). 

The *osseous IP* is hereby defined as the fascial plane between iliopsoas complex and the iliac corpus that cranially extends through iliac ala to iliac crest, with its caudal end being the iliac bony groove bounded laterally by AIIS and medially by iliopectineal eminence (Figure 1 and Figure 2b). Because IM and IT converge inferomedially onto the lesser trochanter of femur, the *ligamentous IP* is defined as the inverted triangle-shaped fascial plane between iliopsoas complex and the capsular ligaments of hip and is bounded laterally by IM and medially by IT with its closely associated iliopectineal bursa (Figure 1 and Figure 2b). In contrast, the *muscular IP* is defined as the fascial plane between iliopsoas complex and its laterally associated IM muscle bundle (Figure 1b–d). As the IM grows bigger in size caudally, IP becomes an L-shaped fascial plane formed vertically by the muscular IP and horizontally by the ligamentous IP (Figure 1). The needle tip position of IPB lies at the junction of the muscular and ligamentous IP (Figure 1c) [7].

### 2.4. Fascia Iliaca Compartment (FIC) and Subpectineal Plane (SP)

Fascia iliaca compartment (FIC) is a well-known potential space superficial to the iliopsoas complex but deep to fascia iliaca per se (Figure 1) and is the target during femoral 3-in-1 block and fascia iliaca compartment block [28]. Fascia iliaca attaches laterally to the ASIS and blends with the fascia covering sartorius muscle, and it is medially continuous with the pectineal fascia. It is covered by fascia lata and forms the roof of a fat-filled space (lacuna musculorum) that contains femoral nerve proper [16,28]. Subpectineal plane (SP) is a loosely defined musculofascial plane between the pectineus muscle and the obturator externus muscle [1,29], into which the anterior and posterior divisions of the obturator nerve enter from the obturator canal [16] (Figure 1d). 

### 2.5. Pericapsular Nerve Group (PENG) Block and Iliopsoas Plane Block (IPB): Same, Same but Different?

The anterior hip capsule has become the main target of hip analgesia because it is now known to be the most richly innervated part of the hip joint [30] that is innervated by the articular branches of femoral nerve, obturator nerve, and accessory obturator nerves [31,32,33,34]. With the goal of achieving sensory block to hip that spares motor involvement, both PENG block [1] and IPB [7,8] were initially developed applying this anatomical knowledge to selectively target the articular branches to anterior hip capsule. 

PENG block, described by Giron-Arango et al. [1], is performed with the probe starting in transverse section over the AIIS. The probe is then rotated clockwise (on the right side) for approximately 45 degrees to align with the pubic ramus. In its original text de-scription, the final needle tip position was said to be placed between IT anteriorly and pubic ramus posteriorly. However, note that because pubic ramus is a pelvic structure medial to the iliopectineal eminence and is not directly deep to IT, the authors were supposed to refer to the iliac corpus or iliopectineal eminence instead of pubic ramus (Figure 1b). Under ultrasound, 20 mL of local anesthetics are then injected for adequate spread within the musculofascial plane. It was proposed to be a true pericapsular block covering the articular branches from femoral nerve, obturator nerve, and accessory obturator nerves [22]. 

Nielsen et al.’s IPB [7,8], on the other hand, was originally developed to block the articular branches of femoral nerve alone. In a letter to editor commenting on PENG block, they further added that branches of accessory obturator nerve may also be covered by IPB [35]. The probe is placed in transverse scan just caudal to the ASIS, rotated 20-30 degrees counterclockwise (on the right side), and parallel shifted along the inguinal ligament until the hip joint is identified where the head of femur dives deep to the acetabular rim. The final needle tip is placed between the iliopsoas complex and iliofemoral ligament lateral to IT (Figure 1c), and 5 mL of local anesthetic is injected into the fascial plane (i.e., IP).

Note that although the method for finding sonographic targets differs between PENG block and IPB, their respective points of injection are actually very close to each other anatomically [23] (Figure 2). Therefore, PENG block and IPB can be recognized as two highly similar fascial plane blocks, both targeting the IP but against different anatomical floors that are just a few centimeters apart [36]. To put it plainly, Giron-Arango et al.’s PENG block is an *osseous IP* injection against the iliac corpus or iliopectineal eminence deep to IT and proximal to the psoas valley (Figure 1b and Figure 2), while Nielsen et al.’s IPB is a *ligamentous IP* injection against the capsular ligaments of hip lateral to IT and distal to the psoas valley (Figure 1c and Figure 2). Regardless of the method, both techniques achieve their analgesic effects by blocking the articular branches of femoral nerve and perhaps also accessory obturator nerve that traverse the IP superomedial to the anterior capsule of hip. With 4 times more injectate volume, PENG block further increases its effects by flooding into the territory of obturator nerve’s articular branches. Both techniques also rely on the spatial isolation of IP by the iliopsoas complex and its associated structures to achieve their proclaimed motor-sparing property. If the injectate can be perfectly restricted within IP, both femoral nerve proper (which resides in FIC), obturator nerve proper (which travels intrapelvically along the pelvic brim just medial to iliopsoas complex), and obturator nerve divisions (which extrapelvically re-sides in SP, and in some cases within obturator externus) can all theoretically be spared, avoiding motor involvement. However, as previously mentioned, although the team has never explicitly revealed its rationale of putting the needle tip directly under IT, PENG block might actually be designed to target the iliopectineal bursa in order to simultaneously block all the articular branches from femoral, obturator, and accessory obturator nerve [22,23]. 

## 3. Methods

We performed a comprehensive search of the literature to look for all published articles relevant to PENG block and IPB using the keyword “pericapsular nerve group” OR “iliopsoas plane” through online peer-reviewed databases PubMed, Embase, and Cochrane Library. Duplications and ongoing trials were first removed from all records returned from the search engines, and then two authors (SRY and YC) manually combed through the titles and abstracts to specifically look for cadaveric and clinical studies utilizing PENG block or IPB. Articles that were conference posters, pediatric studies, applying the two techniques for different purposes other than nerve block, written in non-English languages, or deemed irrelevant for other reasons were excluded. The remaining articles were then assessed in full length for eligibility. Among the selected clinical studies, only articles that specifically documented either the post-procedural motor functions of the quadriceps femoris muscle or the loss of sensation in dermatomes corresponding to the femoral nerve, suggesting extra-IP injectate spread into the FIC, without supplementation of other nerve blocks that can affect quadriceps functions were included for discussion. The last search was performed on 15 July 2022.

For each selected clinical study, the following data were extracted: last name of the first author, time of publication, country of origin, study type, case number, the final needle tip position before injection with its position in the IP and relative to the IT, bolus volume, quadriceps weakness frequency (when available), the occurrence of extra-IP injectate spread (when deducible), and the routes of injectate spread (when deducible). The final needle tip position, the existence of extra-IP spread, and the corresponding route of spread were deduced from the study description or published images/videos. Selected studies were then categorized into 3 groups based on how post-procedural motor blocks were manifested: (A) studies that reported quadriceps weakness, (B) studies with outcomes suggesting potential quadriceps weakness (from direct and indirect evidence of extra-IP injectate spread into the FIC), and (C) studies that specifically reported no occurrence of quadriceps weakness. When deducible, extra-IP spread into the SP is also shown.

## 4. Results

**Figure 3:**
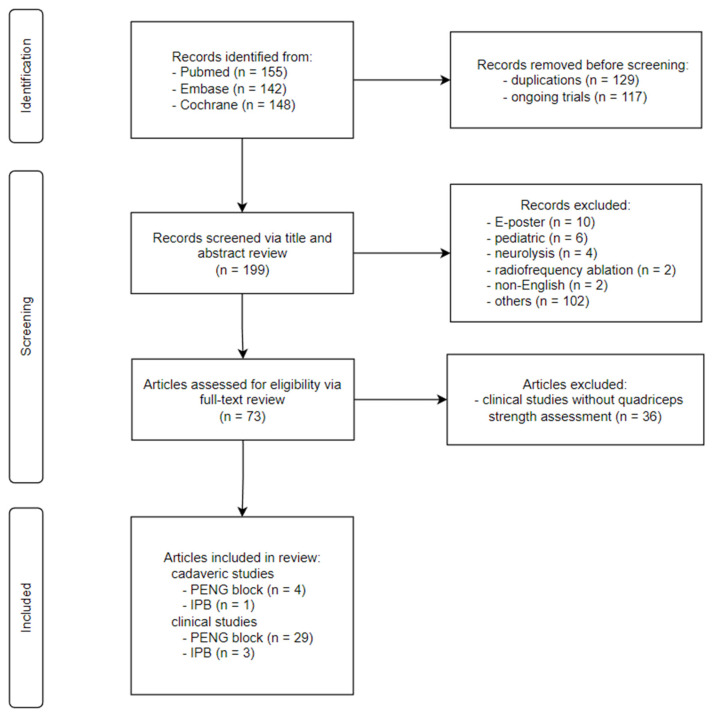
Flowchart for the selection of studies for the current review. IPB: iliopsoas plane block; PENG: pericapsular nerve group.

After article identification and screening, 73 studies applying either PENG block or IPB were reviewed in full length. Among these studies, 36 of them were clinical studies investigating the analgesic effects but without an assessment of quadriceps function and were excluded. Finally, we found 5 cadaveric dye injection studies and 32 clinical studies to be included in our review (Figure 3). Among the cadaveric studies, there are four articles on PENG block [22,37,38,39] and one on IPB [8]. All clinical studies except one randomized controlled trial (RCT) [7] and two recently published case series [40,41] utilized PENG block. 

Among clinical studies utilizing PENG block (*n* = 29), most are low-quality case reports/series [1,2,3,42,43,44,45,46,47,48,49,50,51,52,53,54,55,56,57,58], but one cohort study [59] and eight randomized controlled trials [4,5,60,61,62,63,64,65] appeared in the literature after late 2020. Definite post-procedural quadriceps weakness after PENG block was reported in two case reports [2,3] and two RCTs [4,5] (group A). Among PENG block studies that reported no post-procedural quadriceps weakness or made no mention of motor function at all, clinical manifestations of extra-IP spread of local anesthetics either as observed injectate spread to the FIC in ultrasound images [42], sensory loss of dermatomes corresponding to the femoral nerve [43,44,45,46,47,49,50,51], or an absence of statistically significant difference in quadriceps strength in patients receiving a supra-inguinal fascia iliaca compartment block (SI-FICB) [60,61] were found in nine case reports/series [42,43,44,45,46,47,49,50,51] and two RCTs [60,61] (group B). Nine case reports/series [1,48,52,54,55,56,57,64,66], one cohort study [60], and four RCTs [62,63,64,65] using PENG block specifically reported no motor block at all (group C). 

Among clinical studies utilizing IPB (*n* = 3), the volunteer RCT [7] reported no statistically significant post-procedural quadriceps weakness, but there was radiologic evidence of minor injectate spread into the FIC (group B). The two most recent case series using IPB [40,41] reported no post-procedural motor block at all (group C). The complete list of included studies and their characteristics are shown in Table 1.

**Table 1:** Characteristics of the included clinical studies. See Appendix A for a description of relevant findings from these studies.

First Author (Published Date, Country)	Study Type (Case Number)	Final Needle Tip Position		Bolus Volume	Quadriceps Weakness Frequency	Extra-IP Injectate Spread ^†^	Routes of Injectate Spread
		Position in IP	Relative to IT				
A. Studies reporting quadriceps weakness (PENG block, *n* = 4)							
Yu [2] (May 2019, Canada) ^a^	Case report (2)	Medial border of osseous IP (PENG block)	Deep to IT but more cephalad and superficial (case 1), more medial (case 2)	20 mL	<2% (purported, no supporting data)	FIC (+)	Medial fascial plane (bursal) spread, superficial intramuscular spread
Ahiskalioglu [3] (May 2020, Turkey) ^b^	Case report (2)	Medial border of osseous IP (PENG block)	Not specified	30 mL	Not assessable	FIC (+), SP (+)	Not deducible
Lin [5] (February 2021, Australia) ^c^	RCT (30)	Medial border of osseous IP (PENG block)	Deep to IT	20 mL	26%	FIC (+)	Medial fascial plane (bursal) spread
Aliste [4] (July 2021, Chile) ^d^	RCT (20)	Medial border of osseous IP (PENG block)	Deep to IT	20 mL	25–45% ^¶^	FIC (?), SP (?) ^¶^	Not deducible
B. Studies with signs of potential quadriceps weakness (PENG block, *n* = 11; IPB, *n* = 1)							
Santos [42] (June 2019, Portugal) ^e^	Case report (1)	Medial border of osseous IP (PENG block)	Deep to IT	20 mL	-	FIC (+) ^‡^, SP (−) ^‡^	Medial fascial plane (bursal) spread, lateral fascial plane spread
Aydin [43] (August 2019, Turkey) ^f^	Case report (2)	Medial border of osseous IP (PENG block)	Deep to IT	30 mL	-	FIC (+), SP (+)	Not deducible
Nielsen [7] (October 2019, Denmark) ^g^	RCT (20), volunteer study	Junction between ligamentous and muscular IP (IPB)	Lateral to IT	5 mL	-	FIC (+), SP (−)	See Figure 4
Ahiskalioglu [45] (February 2020, Turkey) ^h^	Case report (1)	Medial border of osseous IP (PENG block)	Deep to IT	30 mL	-	FIC (+), SP (+)	Not deducible
Ahiskalioglu [44] (March 2020, Turkey) ^i^	Case report (2)	Medial border of osseous IP (PENG block)	Deep to IT	30 mL	-	FIC (+), SP (+)	Not deducible
Sandri [46] (June 2020, Italy) ^j^	Case series (10)	Medial border of osseous IP (PENG block)	Not specified	40 mL	-	FIC (+), SP (+)	Not deducible
Talawar [47] (July 2020, India) ^k^	Case report (1)	Medial border of osseous IP (PENG block)	Deep to IT	20 mL	-	FIC (+)	Not deducible
Singh [49] (October 2020, India) ^l^	Case report (1)	- ^l^	Superficial to IT (intramuscular)	15 mL	-	FIC (+), SP (+)	Not deducible
Oksuz [50] (March 2021, Turkey) ^m^	Case report (1)	Medial border of osseous IP (PENG block)	Deep to IT	35 mL	-	FIC (+)	Not deducible
Gong [51] (October 2021, China) ^n^	Case series (5)	Medial border of osseous IP (PENG block)	Not specified	30 mL	-	FIC (+)	Not deducible
Choi [60] (March 2022, Korea) ^o^	RCT (27)	Medial border of osseous IP (PENG block)	Deep to IT	20 mL	-	FIC (+) ^§^	Medial fascial plane (bursal) spread
Senthil [61] (March 2022, India) ^p^	RCT (20)	Medial border of osseous IP (PENG block)	Deep to IT	30 mL	-	FIC (+) ^§^	Not deducible
C. Studies reporting no quadriceps weakness (PENG block, *n* = 14, IPB, *n* = 2)							
Giron-Arango [1] (November 2018, Canada)	Case series (5)	Medial border of osseous IP (PENG block)	Deep to IT	20 mL	-	-	Medial fascial plane (bursal) spread
Mistry [52] (March 2019, India)	Case series (5)	Medial border of osseous IP (PENG block)	Deep to IT	no information	-	-	Medial fascial plane (bursal) spread, lateral fascial plane spread
Pagano [55] (December 2019, Italy)	Case series (6)	Medial border of osseous IP (PENG block)	Not specified	20 mL	-	-	Lateral fascial plane spread
Prado-Kittel [58] (March 2020, Chile)	Case report (1)	Medial border of osseous IP (PENG block)	Deep to IT	20 mL	-	-	Medial fascial plane (bursal) spread
Casas Reza [56] (April 2020, Spain)	Case series (8)	Medial border of osseous IP (PENG block)	Not specified	20 mL	-	-	Not deducible
Alrefaey [62] (September 2020, Egypt)	RCT (30)	Medial border of osseous IP (PENG block)	Not specified	20 mL	-	-	Medial fascial plane (bursal) spread
Singh [48] (September 2020, India)	Case series (10)	Medial border of osseous IP (PENG block)	Deep to IT	20 mL	-	-	Medial fascial plane (bursal) spread
Fujino [57] (March 2021, Japan)	Case report (2)	Medial border of osseous IP (PENG block)	Deep to IT	20 mL	-	-	Medial fascial plane (bursal) spread, lateral fascial plane spread
Rocha-Romero [54] (April 2021, Costa Rica)	Case series (5)	Medial border of osseous IP (PENG block)	Deep to IT	20 mL	-	-	Not deducible
Pascarella [65] (May 2021, Italy)	RCT (30)	Medial border of osseous IP (PENG block)	Deep to IT	20 mL	-	-	Medial fascial plane (bursal) spread
Allard [59] (June 2021, France)	Cohort study (21)	Medial border of osseous IP (PENG block)	Deep to IT	20 mL	-	-	Not deducible
Hua [63] (February 2022, China)	RCT (27)	Medial border of osseous IP (PENG block)	Deep to IT	20 mL	-	-	Not deducible
da Costa [66] (March 2022, Brazil)	Case report (1)	Medial border of osseous IP (PENG block)	Deep to IT	15 mL	-	-	Not deducible
Zheng [64] (March 2022, China)	RCT (36)	Medial border of osseous IP (PENG block)	Deep to IT	20 mL	-	-	Medial fascial plane (bursal) spread
Wang [40] (April 2022, China)	Case series (8)	Junction between ligamentous and muscular IP (IPB)	Lateral to IT	10 mL	-	-	Not deducible
Wang [41] (May 2022, China)	Case series (5)	Junction between ligamentous and muscular IP (IPB)	Lateral to IT	10 mL	-	-	Not deducible

FIC: fascia iliaca compartment; IP: iliopsoas plane; IPB: iliopsoas plane block; IT: iliopsoas tendon; PENG: pericapsular nerve group; RCT: randomized controlled trial; SP: subpectineal plane. † Deduced from clinical observation of quadriceps weakness, adductor weakness, loss of sensation in the corresponding dermatomes, or successful anesthesia of a specific surgical area, unless otherwise specified. For detailed information, please refer to Appendix A. ^‡^ Radiologic evidence of extra-IP spread is also available. ^¶^ extra-IP spread is possible, but residual effects of spinal anesthesia cannot be ruled out. ^§^ There was no statistically significant difference in mean post-operative quadriceps strength between patients receiving PENG block and supra-inguinal fascia iliaca block (SI-FICB), indicating potential extra-IP spread to FIC after PENG block.

**Figure 4:**
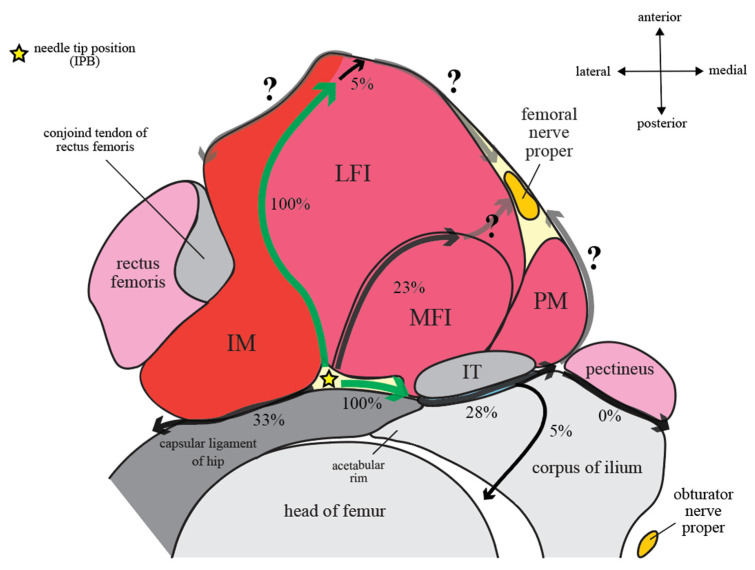
The injectate spread behavior after 5 mL iliopsoas plane block (IPB): Solid arrowed lines represent the routes of spread observed in Nielsen et al.’s IPB volunteer trial [7], while the faded arrowed lines represent the theoretical routes of spread as the injectate volume is increased. Each solid arrowed line is also shown with its respective occurrence frequency observed in the trial [7]. The predominant route of spread (100%) is along the L-shaped iliopsoas plane (IP), comprised of a horizontal fascial plane (i.e., the ligamentous IP) and a vertical raphe between lateral fibers of iliacus (LFI) and iliacus minor (IM) (i.e., the muscular IP), and is colored in green. Lateral fascial plane spread between IM and capsular ligaments of hip toward the gluteus muscles, medial fascial plane spread into the iliopectineal bursa, iliopsoas intramuscular spread along the septum between medial fibers of iliacus (MFI) and LFI, superficial spread along the muscular IP to the anterior surface of iliopsoas complex, and intra-articular spread into hip joint via the bursal-synovium communication were observed in 33%, 28%, 23%, 5%, and 5% of the subjects, respectively. However, definite femoral nerve proper involvement was not readily observed. IT: iliopsoas tendon, PM: psoas major.

## 5. Discussion

For any specific regional block, cadaveric studies are fundamental to understanding its exact mechanism. Therefore, we start the discussion with what can be learned from our included cadaveric studies after PENG block and IPB. However, limitations in the cadaveric dye injection studies are manyfold, and caution should always be exercised when translating its results into living patients. The discussion is then followed by the findings from our included clinical studies and ends with a summary of what we found in a model of injectate spread behavior to explain the mechanism of potential quadriceps weakness after PENG block.

### 5.1. Cadaveric Studies

Early in 2017, in search of a way to reliably block the articular branches of obturator nerve to hip joint, Nielsen et al. found a 15 mL cadaveric dye injection into the SP could consistently block all the articular branches of obturator and accessory obturator nerve to hip joint capsule [29]. They subsequently published another cadaveric study, in which IPB was coined, as they demonstrated the possibility to selectively block the articular branches of femoral nerve to hip joint [8]. After injecting 5 mL of dye into the fascial plane between iliopsoas complex and iliofemoral ligament (i.e., the ligamentous IP), the injectate was found to be contained within a well-defined narrow compartment that they termed IP (Figure 1). Notably, unintentional iliopectineal bursal injection was also found in 27% of the dissections, leading to bursal rupture and the resultant involvement of some of the motor branches from femoral nerve [8]. This observation led to the recommendation of final needle tip positioned more laterally in the IP (Figure 1c) to avoid incidental iliopectineal bursal injection [8].

**Figure 5:**
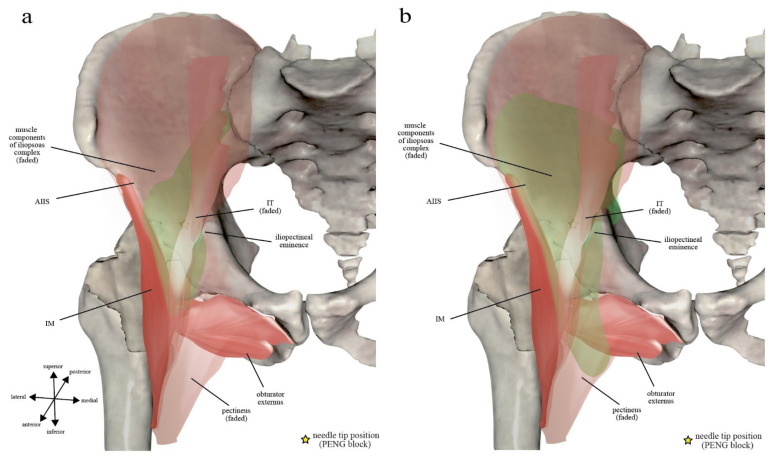
Injectate spread in a cadaver after pericapsular nerve group (PENG) block: (**a**) Injectate spread after 10 mL PENG block is colored in faded green and is overlayed with the faded iliopsoas complex to demonstrate its spatial relationship to the iliacus minor (IM) and iliopsoas tendon (IT). Note the presence of injectate spread deep and medial to IT; (**b**) Injectate spread after 20 mL PENG block is again colored in faded green and overlayed with the iliopsoas complex. In comparison to 10 mL PENG block, the more extensive spread of a 20 mL PENG block includes an inferomedial extension into the subpectineal plane (SP), from where divisions of the obturator nerve emerge, and a superomedial extension into the lesser pelvis, where the obturator nerve proper traverses intrapelvically towards the obturator canal. The final needle tip position of PENG block is marked as a yellow star deep to IT. The illustrated Figure was made from an image acquired from the VH Dissector with permission from Touch of Life Technologies Inc (www.toltech.net), based on the results of a cadaveric dye injection study by Tran et al. [22]. The original image was reconstructed from real cadaver by the Visible Human Project® of National Library of Medicine. AIIS: anterior inferior iliac spine.

After the publication of PENG block by Giron-Arango et al. in late 2018 [1], a dye injection study was soon performed in a lightly embalmed cadaver by the same group to prove its pericapsular coverage [22]. In contrast to IPB, in which only the articular branches of femoral nerve (and perhaps also the accessory femoral nerve) were targeted, the injectate spread in this single cadaver showed that PENG block could simultaneously cover all the articular branches of femoral, obturator, and accessory obturator nerves [22]. They found that 10 mL of dye injected into the fascial plane between IT and iliac corpus could stain the entire anterior hip capsule with minor cranial extension into the pelvis (Figure 4a) [22]. In comparison, 20 mL of dye injected into the same plane on the other side produced more extensive intrapelvic spread cranially up to the level of the ASIS and medially into the lesser pelvis [22] (Figure 5b), potentially reaching the obturator groove to involve the obturator nerve proper intrapelvically [67]. 

When SP lies in close proximity to IP, it is unclear whether these two fascial planes communicate. In the cadaveric study where SP was defined by Nielsen et al., no evidence of inter-planar spread from the SP to IP was found when 15 mL of dye was injected subpectineally [29]. Additionally, when 5 mL of dye was injected into the ligamentous IP (i.e., IPB), no inter-planar spread from the IP to SP was observed either [8]. Therefore, Nielsen and Bendtsen argued that the iliopectineal bursa, which is consistently tightly adherent to the iliofemoral ligament and IT, prevents the inter-planar exchange of the injectate between SP and IP [35]. The iliopectineal fascia, a thickened portion of the fascia iliaca between ASIS and iliopectineal eminence [68], has also been postulated as a partial anatomical barrier between SP and IP [3]. However, after both 10 mL and 20 mL PENG blocks, prominent stain marks could be seen along the undersurface of IT [22], with a staining pattern that matches well with an iliopectineal bursal injection [69]. Intra-articular spread of the injectate into the synovial sac of hip, probably via bursa–synovium communication, was also shown after PENG block [38]. Moreover, the 20 mL side also showed a certain degree of subpectineal staining [22] (Figure 5b). As a result, we speculate that inter-planar spread from IP to SP does occur during PENG block from an incidental iliopectineal bursa injection and its following bursal rupture and/or puncture. More importantly, if PENG block does frequently end up as an iliopectineal bursa injection with potential bursal rupture/puncture, the injectate can then manage to breach the medial border of IP (formed by IT and its closely associated iliopectineal bursa) and subsequently spread anteromedially along the surface of PM to catch the femoral nerve proper within FIC, causing quadriceps weakness. In support of our speculation, a recently published surgeon-performed PENG block cadaver study showed that femoral nerve proper and obturator nerve divisions were stained in 5.6% and 11.1% of their specimen, and abundant dye could again be seen spreading along the undersurface of IT (corresponding to iliopectineal bursa) and further extended anteromedially to involve femoral nerve proper and posteromedially to obturator nerves (extrapelvic) [39]. 

### 5.2. Clinical Studies

PENG block’s clinical effectiveness as a feasible regional analgesic technique for hip, as summarized by a previous scoping review of its analgesic effects on earlier low-quality case reports and letters [70], has continued to be supported by the more recent RCTs. However, direct and indirect clinical evidence of quadriceps weakness after PENG block has also continued to emerge (Table 1). 

Direct evidence first came from Yu et al. [2] in a letter describing two cases of clinically significant quadriceps weakness after the “standard” 20 mL PENG block and acknowledged the needle tip position as a major contributing factor. In one of the most recent RCTs, Lin et al. [5] found an alarmingly high percentage of post-operative quadriceps weakness (26%) in patients receiving a pre-operative PENG block after excluding a case with residual effects of spinal anesthesia. In support of that, Aliste et al. [4] found an even higher percentage of quadriceps weakness (25–45%) after a post-operatively performed PENG block, although some of these motor blocks could be a result of the residual effects of spinal anesthesia. 

Indirect evidence indicating femoral nerve proper involvement after a 20 mL PENG block, deduced either from a loss of sensation in the femoral nerve dermatome, comparison of post-operative quadriceps muscle strength with patients receiving SI-FICB, or the published study images/videos, is ample (Table 1). In a notable case report, although the author did not test for quadriceps motor functions, the femoral nerve proper was clearly shown to be submerged in local anesthetics that dispersed from the injection site deep to the IT anteromedially to the anterior surface of the PM [42]. While most of these studies are low-quality case reports, two recent RCTs also showed hints of a potential post-operative motor block. Choi et al. [60] and Senthil et al. [61] both compared the post-operative quadriceps muscle strength after PENG block and SI-FICB and found no statistically significant difference between these two groups. Since a successful SI-FICB necessitates blocking the femoral nerve proper, their results implied that femoral nerve proper involvement after PENG block might be more frequent than previously expected.

Some interesting evidence of extra-IP spread came from case reports applying high-volume (30 mL and more) PENG blocks, first theorized by Ahiskalioglu et al. [3] to approximate the effects of the femoral 3-in-1 block or even the lumbar plexus block. They performed PENG blocks with 30 mL of local anesthetics and successfully achieved surgical anesthesia for saphenous varicose vein ligation and stripping [3] and medial thigh tumor resection [45], and PENG block served as an alternative to the obturator nerve block in preventing adductor muscle spasms during bladder surgery [44]. Later, another group combined a 35 mL PENG block with a sciatic nerve block to provide surgical anesthesia of the distal tibia and fibula and to cover tourniquet pain in a patient [50]. In a similar case series, a 30 mL PENG block was also combined with a sacral plexus block to provide near-complete anesthesia for minimally percutaneous invasive internal fixation of the femoral neck in five patients [51]. Sandri et al. [46] applied an even higher volume (40 mL) PENG block, supplemented with 10 mL local infiltration of the skin and subcutaneous tissue (performed by a surgeon) and light-to-moderate propofol infusion, and successfully anesthetized five patients for total hip replacement via the direct anterior surgical approach (“bikini” skin crease incision) [71]. 

One may wonder how an interfascial plane block originally designed to selectively block the articular branches to anterior hip capsule would eventually evolve into a block that has managed to provide anesthesia for surgeries requiring extensive sensory coverage. Obviously, total hip replacement surgery necessitates blocking much beyond the sensory territories innervated by the articular branches of femoral, obturator, and accessory obturator nerves. Even when an adequate blockade of femoral nerve proper and obturator nerves is supplemented with a sacral plexus block, blocking the subcostal/iliohypogastric and lateral femoral cutaneous nerves is still needed for a total hip arthroplasty [72]. As a result, these case reports utilizing high-volume PENG block for surgical anesthesia serve as yet another hint that extra-IP spread does occur after PENG block. However, possibilities of technical failure, such as structural misinterpretation during block performance, should also be considered in these low-quality PENG block studies. For example, as pointed out recently by a correspondence letter [73], a case report claiming to have achieved surgical anesthesia for total hip arthroplasty with just a 15 mL PENG block combined with propofol infusion had actually performed a bona fide intramuscular injection (with the needle tip placed directly on top of IT) inside the iliopsoas complex that spread to femoral, obturator, lateral femoral cutaneous, and genitofemoral nerve [49].

IPB, on the other hand, has not received as much attention as PENG block despite the fact that the team has carried out a well-designed study that provides highly valuable radiologic information concerning injectate spread and its technical implication in achieving a true motor-sparing hip block. After the IPB cadaveric study [8], the authors performed a volunteer RCT to investigate the motor functions of femoral and obturator nerves after IPB [7]. Bilateral low-volume (5 mL) ligamentous IP injection lateral to IT was performed in 20 healthy volunteers, one side with local anesthetics and the other side with normal saline. The maximal force in knee extension and hip adduction showed no statistically significant difference between the blocked side and the sham side an hour after IPB. Compared to the significantly more popular PENG block, IPB therefore turned out to be the hip block that is actually motor-sparing. However, IPB injects just one-fourth of a “standard” PENG block’s volume and theoretically lacks the articular coverage of obturator nerve, so its analgesic effects await further clinical proof. Just recently, two case series that adopted IPB for the first time in patients receiving hip surgeries were published. A 10 mL IPB was performed pre-operatively to look for its analgesic effects and side effects in quadriceps involvement, and their results supported IPB’s claim as an effective sensory block to hip that is also motor-sparing [40,41]. Nonetheless, higher-quality clinical studies, especially ones with a head-to-head comparison with PENG block, are needed for IPB. 

### 5.3. Injectate Spread Behavior

Whether PENG block and IPB can be used as an effective sensory block to hip that is also motor-sparing is determined by how injectates spread along and outside the IP. While there is no definitive radiologic evidence currently available for PENG block, the IPB volunteer study has given us magnetic resonance images of how injectates behave within the IP fascial continuity in living human bodies [7]. Given the high similarities between PENG block and IPB, the injectate spread behavior observed in IPB (Figure 5) may serve as a template for us to make speculations on how injectates behave along the IP during PENG block. 

#### 5.3.1. Craniocaudal Spread within Iliopsoas Plane (IP)

According to the IPB trial [7], the most consistently observed route of spread was within the narrow compartment of IP (100%). The typical spread after 5 mL IPB follows a craniocaudal direction along the IP and is spatially restricted within an L-shaped anatomical channel deep to iliopsoas complex that is bordered laterally by IM and medially by IT with its closely associated iliopectineal bursa (Figure 5). The channel floor is formed superiorly by the iliac bony groove between AIIS and iliopectineal eminence and inferiorly by the ligamentous trough between iliofemoral ligament and pubofemoral ligament of the capsular ligaments of hip [38] (Figure 3). Following this channel, a 5 mL IPB injection was shown to result in a well-defined spread along the IP cranially up to iliac ala and caudally down to the level of lesser trochanter [7] (Figure 4). 

**Figure 6:**
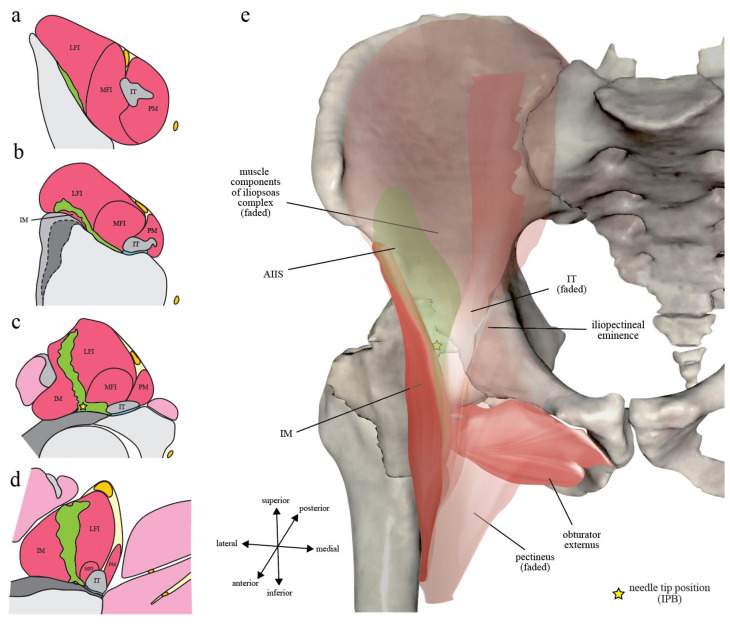
Injectate spread in living human subjects after iliopsoas plane block (IPB): (**a**–**d**) The most commonly observed pattern of injectate spread after 5 mL IPB, illustrated as the green-colored area, is superimposed on the four transverse section levels as depicted in Figure 1; (**e**) The injectate spread is colored in faded green and is overlayed by the iliopsoas complex to demonstrate its spatial relationship to iliacus minor (IM) and iliopsoas tendon (IT). Note that the injectate is confined within a well-defined iliopsoas plane (IP) without the extra-IP spread that is deep and medial to IT as in PENG block (Figure 6). However, there is superficial spread via the muscular IP towards fascia iliaca compartment (FIC) (**c**,**d**), potentially reaching femoral nerve proper when given higher volume of injection. The illustrated Figure was made from an image acquired from the VH Dis-sector with permission from Touch of Life Technologies Inc (www.toltech.net), based on the magnetic resonance images by Nielsen et al. [7]. The original image was reconstructed from real cadavers by the Visible Human Project® of National Library of Medicine.

Because of the IP fascial continuity, it seems logical that injectate spread after PENG block may initially (at least during the first 5–10 mL injection) also follow a similar pattern to IPB. But as previously discussed, the medial needle tip position of PENG block (at the medial border of osseous IP, directly deep to IT) may easily result in iliopectineal bursa injection instead of a true IP injection as in IPB. And with a much larger injecting volume, PENG block naturally results in more extensive and less well-defined spread, especially when the bursa is ruptured by volume or pressure overload and/or is punctured by the needle tip, than the low-volume IPB (Figure 5 and Figure 6).

#### 5.3.2. Spread Outside of Iliopsoas Plane (IP): Extra-IP Spread

Alternative routes of spread observed in the IPB trial [7] include lateral spread be-tween IM and capsular ligament of hip, medial spread into iliopectineal bursa, intramuscular spread along the MFI-LFI septum of iliopsoas complex, intra-articular spread into the hip synovium, and superficial spread to the anterior surface of iliopsoas complex (i.e., FIC) (Figure 6). It is important to note that even as the needle tip was deliberately placed lateral to IT during IPB, the medial spread into iliopectineal bursa still occurred in 28% of the subjects, an occurrence rate very similar to what was observed in cadavers (27%) [8]. And with an injection volume even as low as 5 mL, FIC was still breached in 5% of the cases. Besides, although not officially reported by the authors, superficial intramuscular spread along the MFI-LFI septum of iliopsoas complex could clearly be seen in 23% of the volunteer subjects [7]. As the volume of injection is further increased, it is likely that femoral nerve proper can be flooded via at least one of these routes.

**Figure 7:**
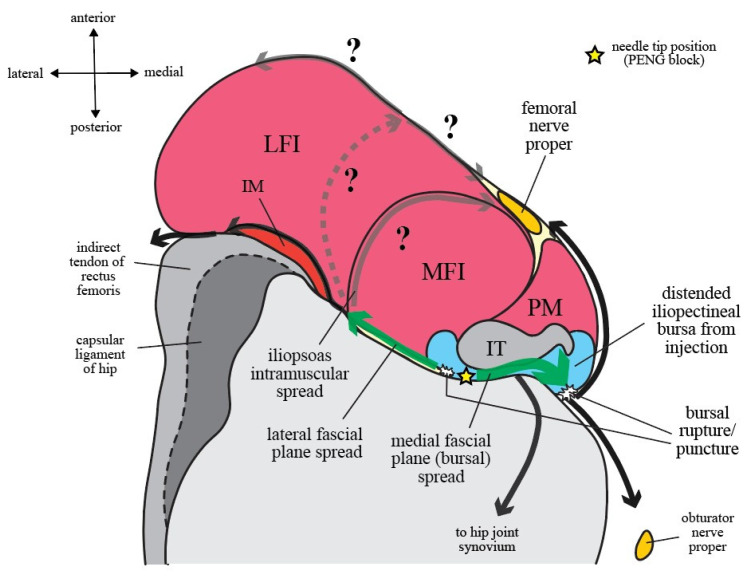
The injectate spread behavior after 20 mL pericapsular nerve group (PENG) block: Solid arrowed lines represent the routes of spread that have been observed in the cadaveric or clinical studies included in this review, while the faded arrowed lines represent theoretical routes of spread that await further evidence. The dashed arrowed line marks the superficial spread via the muscular iliopsoas plane (IP) as in Figure 5. Medial fascial plane (bursal) spread is the most commonly observed route of spread under ultrasound after PENG block (Table 1). Since Iliopectineal bursa lies immediately deep to iliopsoas tendon (IT), it can be easily injected during PENG block. As the bursa ruptures from pressure/volume overload or is punctured by the needle tip, both the anteromedial spread along the anterior surface of psoas major (PM) to involve femoral nerve proper and the posteromedial spread to involve either the obturator nerve proper (intrapelvic) or its divisions (extrapelvic) can occur [22,39]. When iliopectineal bursa is injected, intra-articular spread via the bursal-synovium communication can also occur [38]. Lateral fascial plane spread is the second most commonly observed spread route after PENG block (Table 1), and a further continuation of this lateral spread to the gluteus muscles were shown in another cadaveric study [37].

Based on the injectate spread behavior observed in IPB (Figure 4), we then produced a model of injectate spread behavior after PENG block (Figure 7) according to the deduced routes of spread from the currently available PENG block clinical studies (Table 1). After PENG block, the most frequently observed route of spread was medial fascial plane (bursal) spread [1,2,5,42,48,52,57,58,60,62,64,65], followed by lateral fascial plane spread [42,52,55,57] and superficial intramuscular spread of the iliopsoas complex [2] (Table 1). Note that the frequency of superficial intramuscular spread is very likely to be underestimated since its occurrence could not be readily distinguished from the static images provided by these studies. 

The medial fascial plane spread deep to IT was the most commonly observed route of spread during PENG block (Figure 7). In fact, Giron-Arango et al. viewed this medial spread under IT during PENG block as technically desirable [6] because the injectate can then spread medially to cover the articular branches of obturator and accessory obturator nerves for a more comprehensive block of the anterior hip capsule. However, this medial fascial plane spread seen on ultrasound is likely the result of a direct iliopectineal bursal injection. In an iliopsoas peritendinous injection study under ultrasound for diagnosing tendinosis and bursitis, the authors learned that needle placement deep to IT at the level of iliopectineal eminence would readily fill the iliopectineal bursa, allowing them to reduce the amount of needle manipulation for successful bursal entry [74]. More recently, an ultrasound-guided iliopectineal bursa contrast injection study performed a precise injection into the bursa at the acetabular rim deep to the lateral aspect of IT, and the injectate was found to distend the bursa into a well-defined U-shaped sac that lifts up IT and stretches craniocaudally along the IT’s undersurface [69]. In addition, a peritendinous space deep to IT that is both outside the bursa and amenable to injection does not seem to exist [69]. As a result, placing the needle tip deep to IT on the iliac corpus (following PENG block’s original method) would probably almost always result in a direct iliopectineal bursal injection and can be observed on ultrasound as medial fascial plane spread that lifts up IT.

One of the most commonly speculated mechanisms of quadriceps weakness after PENG block is the anteromedial spread of local anesthetics along the surface of PM to capture femoral nerve proper within FIC [67]. It has been argued that this anteromedial spread occurs from a needle tip placed either too superficially (intramuscular injection) or too medially relative to IT [2,18,67]. However, instead, we speculate that this medial extra-IP spread results from either a ruptured bursa by volume/pressure overload, a punctured bursa during PENG block’s needle maneuvering, or most probably both, even if the needle tip is placed “optimally” between IT and iliac corpus. When the iliopectineal bursa is ruptured and/or punctured, the medial limit of IP will no longer be intact, and extra-IP spread will occur. Medial fascial plane spread can then not only continue anteromedially to the femoral nerve proper within FIC along the surface of PM but also spread posteromedially to the obturator nerve divisions within SP (extrapelvic) or deep to the obturator nerve proper along the pelvic brim (intrapelvic) (Figure 7). Therefore, as PENG block may be a true pericapsular block to the anterior hip capsule via this medial extra-IP spread to cover the articular branches of femoral, obturator, and accessory obturator nerve, its concurrent spread to the femoral nerve proper within FIC that leads to quadriceps weakness is probably unavoidable, especially when given a higher-volume injection.

The lateral fascial plane spread along the osseous IP was the second most commonly observed route of spread after PENG block (Figure 7) and can also occur after IPB (Figure 6). Staining of the gluteus muscles was observed in a cadaveric dye injection study after 30 mL PENG block [37], which probably resulted from a lateral continuation of this lateral fascial plane spread between LFI and the indirect tendon of rectus femoris and capsular ligaments of hip (Figure 7). Moreover, since involvement of the lateral femoral cutaneous nerve was already reported in several case reports after PENG block [3,42,44,45,49,50], a superficial continuation of this spread via the muscular IP to FIC that further diverges laterally might also exist, especially when the injection volume is large enough. As it happens, its medial divergence can also spread to involve femoral nerve proper (Figure 7).

The superficial intramuscular spread along the MFI-LFI septum of iliopsoas complex occurred in about one-fourth of the volunteer subjects in the IPB trial [7] (Figure 7). Since femoral nerve proper resides just superficial to the MFI-LFI septum in FIC (Figure 6 and Figure 7), it is not surprising that it is occasionally captured by the injectate during higher-volume PENG block. It has also been postulated that the injectate may track along the articular branches intramuscularly back to femoral nerve proper [75]. However, again, more imaging evidence during PENG block is needed to confirm these speculations. 

### 5.4. Factors Leading to Quadriceps Weakness

Three important factors can contribute to PENG block’s extensive and sometimes superfluous spread (Figure 5) in comparison to IPB’s well-defined spread restricted within the IM-IT channel (Figure 6). 

Firstly, the few centimeters of difference in the final needle tip position between PENG block and IPB (Figure 2) may play a significant role in determining the chance of incidental iliopectineal bursal injection. Because the needle tip of PENG block is placed directly deep to IT (Figure 7), compared to IPB’s more laterally placed needle tip at the junction of the ligamentous IP and muscular IP (Figure 4), the iliopectineal bursa can be more easily injected during PENG block. When the bursa is ruptured by injectate or is simply punctured on its deep surface, the following extra-IP spread of injectate can flood anteromedially to capture the femoral nerve proper within FIC, causing unwanted quadriceps weakness.

Secondly, the high injection volume adopted in PENG block, which is at least four times that of IPB, naturally results in more extensive spread. The increase in injection volume seems to initially result in craniocaudal spread (as in 10 mL PENG block) (Figure 5a) and later by lateromedial spread (as in 20 mL PENG block) (Figure 5b), implying an initial intra-bursal spread followed by a bursal rupture at a larger volume due to volume overload. Not surprisingly, a volume reduction to 5 to 10 mL has recently been advocated for PENG block to avoid inadvertent quadriceps weakness [75].

Thirdly, since the iliopectineal bursa is a finite space, its rupture can also be caused by pressure overload. A high injection pressure should probably be avoided, especially when the medial fascial plane (bursal) spread lifting the IT is observed on ultrasound during injection. However, further study is needed to define the optimal injection pressure threshold.

### 5.5. Recommendations

We recommend PENG block to be the analgesic technique of choice for the anterior hip capsule only when post-procedural quadriceps weakness is not an immediate clinical concern. For example, during the wait for surgeries of traumatic femoral neck fracture in the emergency room, PENG block performed with short-acting local anesthetics may provide excellent analgesia to the anterior hip capsule due to its extensive pericapsular coverage of the articular branches of femoral, obturator, and accessory obturator nerve, as the patient remains resting in bed. However, IPB should be considered when intact post-procedural motor functions of quadriceps femoris are necessary. For example, as early mobilization has become a vital component of the enhanced recovery after surgery (ERAS) clinical pathway after total hip arthroplasty [76], IPB should be the technique of choice to facilitate achieving early functional recovery by providing adequate analgesia to the anterior hip capsule while avoiding complications such as early prosthetic dislocations from fall due to quadriceps weakness during in-hospital rehabilitation.

Regardless of the method, keeping the needle tip more laterally away from the undersurface of IT, preferably at the junction between osseous/ligamentous IP and muscular IP (Figure 1b,c), may help reduce the risk of incidental iliopectineal bursa injection and its resultant bursal rupture/puncture, which may continue anteromedially to flood the femoral nerve proper within FIC. In addition, it is always prudent to start with a lower volume (5–10 mL). A lower injection pressure should probably also be considered.

## 6. Conclusions

PENG block and IPB are two highly similar ultrasound-guided interfascial plane blocks targeting the IP for analgesia of the anterior hip capsule but differ in their final needle tip position and volume of injection, which may play significant roles in determining their proclaimed motor-sparing property. PENG block places its needle tip directly deep to IT and can frequently end up as an iliopectineal bursal injection that leads to bursal rupture under volume/pressure overload, or bursal puncture during needle maneuvering, or most probably both. The result is a breach of the medial limit of IP formed by IT and its closely associated iliopectineal bursa, causing an extra-IP injectate spread anteromedially to femoral nerve proper within FIC and posteromedially to obturator nerve and its branches. As more clinical studies utilizing PENG block appear in literature, evidence from higher quality trials has shown that PENG block can result in 25% or more of quadriceps weakness post-operatively. In comparison, IPB places its needle tip lateral to IT and inject just one fourth of PENG block’s volume. Its injectate spread has been shown to be restricted inside IP, within a well-defined L-shaped channel bounded laterally by IM and medially by IT and its closely associated iliopectineal bursa. Without the medial extra-IP spread as in PENG block, the currently available clinical data of IPB, albeit still limited, supports it to be the true motor-sparing hip block. 

Accordingly, we recommend that PENG block should only be indicated in clinical scenarios when post-procedural quadriceps weakness is not an immediate concern, for example, during the wait for femoral neck fracture surgeries in the emergency room. When intact motor functions of quadriceps femoris are required, for example, during early mobilization following the ERAS pathway after total hip arthroplasty, IPB should be the analgesic technique of choice. Irrespective of the method, we recommend a more laterally placed final needle tip position within IP, away from the undersurface of IT (where iliopectineal bursa is located), and a lower starting volume (5–10 mL) to reduce the risk of extra-IP injectate spread to the undesired neural targets, especially femoral nerve proper within FIC. Future studies should focus on comparing PENG block and IPB for their analgesic effects and side effects on motor function impairment. 

## Data Availability

Not applicable.

## Appendix A

**Brief summary of the study findings listed in Table 1:**

^a^ Inadvertent quadriceps weakness were observed in two cases out of over 100 PENG blocks performed. In the first case, the block was technically challenging and resulted in a needle insertion point slightly more cephalad and much of the injectate was delivered more superficially than the approach described by Giron-Arango et al. For the second case, the final needle tip location and site of injection was noted to be more medial than the classic description of the PENG block, with the needle tip located on the medial side of the IT.

^b^ The study only presented two cases and deliberately used high-volume PENG block to attempt to flood the femoral and obturator nerve. Hence, no calculated percentage of quadriceps weakness was presented in Table 1. Loss of sensation was observed in dermatomes of femoral, lateral femoral cutaneous, genitofemoral, anterior femoral cutaneous, obturator, and saphenous nerve with significant quadriceps weakness in case 1. Loss of sensation in dermatomes of femoral, obturator, lateral femoral cutaneous, and genitofemoral nerve was also observed in case 2, but motor examination was not performed due to patient’s fracture.

^c^ The study compared pre-operatively performed PENG block to femoral nerve block in patients receiving either general anesthesia or spinal anesthesia for hip fracture surgeries. In the recovery unit, 10 patients (33%) in the PENG block group had quadriceps motor impairment but 2 of them were attributed to residual effects from spinal anesthesia be-cause the weakness were bilateral.

^d^ The study compared post-operatively performed PENG block to SI-FICB in patients receiving total hip arthroplasty after spinal anesthesia. Quadriceps motor impairment was observed in 45% and 25% of the patients in the PENG block group as compared to 90% and 85% of the patients in the SI-FICB group at the 3rd and 6th post-operative hour, respectively. Hip adduction motor impairment was also observed in 50% and 50% of the patients in the PENG block group as compared to 90% and 65% in the SI-FICB group at the 3rd and 6th post-operative hour, respectively. Besides, 10% of the patients had sensory loss of the medial thigh in the PENG block group as compared to 80% in the SI-FICB group at the 6th post-operative hour.

^e^ Femoral nerve involvement was not reported in text but femoral nerve proper was shown to be submerged in local anesthetics in their published figure; analgesia of the territory covered by lateral femoral cutaneous nerve was also noted. SP spread was not observed under X-ray following a post-operative contrast injection via a catheter placed in IP after initial bolus.

^f^ PENG block was used for surgical anesthesia of varicose vein ligation involving derma-tomes of femoral nerve and obturator nerve. Sensory testing of the femoral, obturator, lateral femoral cutaneous, and genitofemoral nerve dermatomes revealed a sufficient level of anesthesia.

^g^ One volunteer out of 20 was found to have a reduction of the maximal force of knee ex-tension in both the blocked and sham side. Minor superficial spread of injectate to FIP was also found in the same subject. Absence of SP spread was specifically confirmed by MRI images.

^h^ PENG block was used for surgical anesthesia of medial thigh tumor resection. Sensory testing of the femoral nerve, obturator nerve, and lateral femoral cutaneous nerve derma-tomes revealed a sufficient level of anesthesia.

^i^ PENG block was used to prevent adductor muscle spasm during transurethral resection of bladder tumor. Post-operative examination revealed loss of sensation in dermatomes of obturator, femoral, lateral femoral cutaneous, and genitofemoral nerve.

^j^ PENG blocks were applied to achieve surgical anesthesia of total hip replacement via the direct anterior approach (“bikini” skin crease incision) [71] under 0.025-0.05 mg/kg/min propofol infusion and 10 mL local infiltration of skin and subcutaneous soft tissue by the surgeon. All 5 patients were able to perform hip flexion-extension ranging from 90 to 120 degree before surgery. However, no information concerning quadriceps femoris motor strength was reported.

^k^ Loss of sensation of anterior and medial compartment of thigh without quadriceps weakness were reported.

^l^ PENG block with 15 mL bolus injection followed by catheter continuous infusion supplemented with propofol sedation was performed to achieve surgical anesthesia of bipolar hemiarthroplasty in a patient. Sensory testing of femoral, obturator, lateral femoral cutaneous, and genitofemoral nerve dermatomes revealed sufficient level of anesthesia. However, it was pointed out by a correspondence letter [73] that the authors actually performed an intramuscular injection of iliopsoas complex due to incorrect structural interpretation of ultrasound image.

^m^ High-volume (35 mL) PENG block was combined with sciatic nerve block to provide anesthesia of distal tibia and fibula fracture surgery and cover thigh tourniquet pain. Dermatomes of femoral, obturator, and lateral femoral cutaneous nerve were evaluated before the start of surgery.

^n^ High-volume (30 mL) PENG block was combined with sacral plexus block, supplemented with on-demand local infiltration, for surgical anesthesia of minimally invasive percutaneous internal fixation of femoral neck.

^o^ The study compared the effects of PENG block and SI-FICB on post-operative quadriceps strength following total hip arthroplasty under general anesthesia. The authors reported the mean post-operative quadriceps muscle strength measured with a dynamometer, and there was no statistically significant difference between the two groups.

^p^ The study compared the effects of post-operative PENG block and SI-FICB on mean quadriceps motor power after spinal anesthesia. There was no statistical difference be-tween the two groups until after 18 hours post-operatively, when patients in the PENG block group started to have slightly better quadriceps motor strength.
