# Correction: Yeoh et al. Pericapsular Nerve Group Block and Iliopsoas Plane Block: A Scoping Review of Quadriceps Weakness after Two Proclaimed Motor-Sparing Hip Blocks. *Healthcare* 2022, *10*, 1565

**DOI:** 10.3390/healthcare10091804

**Published:** 2022-09-19

**Authors:** Shang-Ru Yeoh, Yen Chou, Shun-Ming Chan, Jin-De Hou, Jui-An Lin

**Affiliations:** 1 Department of Anesthesiology, Wan Fang Hospital, Taipei Medical University, Taipei 116, Taiwan; 2 Center for Regional Anesthesia and Pain Medicine, Wan Fang Hospital, Taipei Medical University, Taipei 116, Taiwan; 3 Department of Medical Imaging, Far Eastern Memorial Hospital, New Taipei City 220, Taiwan; 4 Department of Anesthesiology, Tri-Service General Hospital and National Defense Medical Center, Taipei 11490, Taiwan; 5 Department of Anesthesiology, School of Medicine, National Defense Medical Center, Taipei 11490, Taiwan; 6 Division of Anesthesiology, Hualien Armed Forces General Hospital, Hualien 97144, Taiwan; 7 Center for Regional Anesthesia and Pain Medicine, Chung Shan Medical University Hospital, Taichung 40201, Taiwan; 8 Department of Anesthesiology, School of Medicine, Chung Shan Medical University, Taichung 40201, Taiwan; 9 Department of Anesthesiology, Chung Shan Medical University Hospital, Taichung 40201, Taiwan; 10 Department of Anesthesiology, School of Medicine, College of Medicine, Taipei Medical University, Taipei 110, Taiwan; 11 Pain Research Center, Wan Fang Hospital, Taipei Medical University, Taipei 116, Taiwan

In the original publication [1], ***Figures 2, 4, 5 and 6*** were not cited correctly. The citation has now been inserted in ***Discussion***, Injectate Spread Behavior, pages 15–18 and should read:

*5.3. Injectate Spread Behavior*

Whether PENG block and IPB can be used as an effective sensory block to hip that is also motor-sparing is determined by how injectates spread along and outside the IP. While there is no definitive radiologic evidence currently available for PENG block, the IPB volunteer study has given us magnetic resonance images of how injectates behave within the IP fascial continuity in living human bodies [7]. Given the high similarities between PENG block and IPB, the injectate spread behavior observed in IPB (Figure 4) may serve as a template for us to speculate on how injectates behave along the IP during PENG block.

5.3.1. Craniocaudal Spread within Iliopsoas Plane (IP)

According to the IPB trial [7], the most consistently observed route of spread was within the narrow compartment of IP (100%). The typical spread after 5 mL IPB follows a craniocaudal direction along the IP and is spatially restricted within an L-shaped anatomical channel deep to iliopsoas complex that is bordered laterally by IM and medially by IT with its closely associated iliopectineal bursa (Figure 4). The channel floor is formed superiorly by the iliac bony groove between AIIS and iliopectineal eminence and inferiorly by the ligamentous trough between iliofemoral ligament and pubofemoral ligament of the capsular ligaments of hip [38] (Figure 2). Following this channel, a 5 mL IPB injection was shown to result in a well-defined spread along the IP cranially up to iliac ala and caudally down to the level of lesser trochanter [7] (Figure 6).

**Figure 6:**
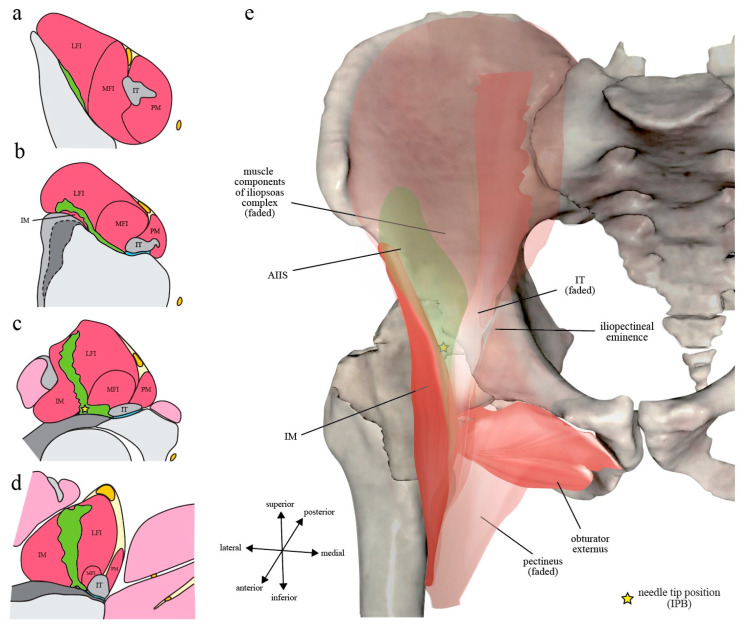
Injectate spread in living human subjects after iliopsoas plane block (IPB): (**a**–**d**) The most commonly observed pattern of injectate spread after 5 mL IPB, illustrated as the green-colored area, is superimposed on the four transverse section levels as depicted in Figure 1; (**e**) The injectate spread is colored in faded green and is overlayed by the iliopsoas complex to demonstrate its spatial relationship to iliacus minor (IM) and iliopsoas tendon (IT). Note that the injectate is confined within a well-defined iliopsoas plane (IP) without the extra-IP spread that is deep and medial to IT as in PENG block (Figure 5). However, there is superficial spread via the muscular IP towards fascia iliaca compartment (FIC) (**c**,**d**), potentially reaching femoral nerve proper when given higher volume of injection. The illustrated figure was made from an image acquired from the VH Dissector with permission from Touch of Life Technologies Inc. (www.toltech.net), based on the magnetic resonance images by Nielsen et al. [7]. The original image was reconstructed from real cadavers by the Visible Human Project^®^ of National Library of Medicine.

Because of the IP fascial continuity, it seems logical that injectate spread after PENG block may initially (at least during the first 5–10 mL injection) also follow a similar pattern to IPB. However, as previously discussed, the medial needle tip position of PENG block (at the medial border of osseous IP, directly deep to IT) may easily result in iliopectineal bursa injection instead of a true IP injection as in IPB. Additionally, with a much larger injecting volume, PENG block naturally results in more extensive and less well-defined spread, especially when the bursa is ruptured by volume or pressure overload and/or is punctured by the needle tip, than the low-volume IPB (Figure 5 and Figure 6).

5.3.2. Spread Outside of Iliopsoas Plane (IP): Extra-IP Spread

Alternative routes of spread observed in the IPB trial [7] include lateral spread between IM and capsular ligament of the hip, medial spread into the iliopectineal bursa, intramuscular spread along the MFI-LFI septum of the iliopsoas complex, intra-articular spread into the hip synovium, and superficial spread to the anterior surface of iliopsoas complex (i.e., FIC) (Figure 4). It is important to note that even as the needle tip was deliberately placed lateral to IT during IPB, the medial spread into iliopectineal bursa still occurred in 28% of the subjects, an occurrence rate very similar to what was observed in cadavers (27%) [8]. Additionally, with an injection volume even as low as 5 mL, FIC was still breached in 5% of the cases. Besides, although not officially reported by the authors, superficial intramuscular spread along the MFI-LFI septum of iliopsoas complex could clearly be seen in 23% of the volunteer subjects [7]. As the volume of injection is further increased, it is likely that femoral nerve proper can be flooded via at least one of these routes.

…

The superficial intramuscular spread along the MFI-LFI septum of iliopsoas complex occurred in about one-fourth of the volunteer subjects in the IPB trial [7] (Figure 4). Since femoral nerve proper resides just superficial to the MFI-LFI septum in FIC (Figure 4 and Figure 7), it is not surprising that it is occasionally captured by the injectate during higher-volume PENG block. It has also been postulated that the injectate may track along the articular branches intramuscularly back to femoral nerve proper [75]. However, again, more imaging evidence during the PENG block is needed to confirm these speculations.

The authors state that the scientific conclusions are unaffected. This correction was approved by the Academic Editor. The original publication has also been updated.
